# Growth Hormone Protects Against Ovariectomy-Induced Bone Loss in States of Low Circulating Insulin-like Growth Factor (IGF-1)*

**DOI:** 10.1359/jbmr.090723

**Published:** 2009-07-13

**Authors:** J Christopher Fritton, Kelly B Emerton, Hui Sun, Yuki Kawashima, Wilson Mejia, Yingjie Wu, Clifford J Rosen, David Panus, Mary Bouxsein, Robert J Majeska, Mitchell B Schaffler, Shoshana Yakar

**Affiliations:** 1 Leni & Peter W. May Department of Orthopaedics, Mount Sinai School of Medicine New York, NY, USA; 2 Department of Medicine, Division of Endocrinology, Diabetes & Bone Disease, Mount Sinai School of Medicine New York, NY, USA; 3 Maine Medical Center Research Institute 81 Research Drive, Scarborough, ME, USA; 4 Orthopedic Biomechanics Laboratory, Beth Israel Deaconess Medical Center, Harvard Medical School Boston, MA, USA

**Keywords:** growth factors, estrogen, apoptosis, remodeling, osteoporosis

## Abstract

Early after estrogen loss in postmenopausal women and ovariectomy (OVX) of animals, accelerated endosteal bone resorption leads to marrow expansion of long bone shafts that reduce mechanical integrity. Both growth hormone (GH) and insulin-like growth factor (IGF-1) are potent regulators of bone remodeling processes. To investigate the role of the GH/IGF-1 axis with estrogen deficiency, we used the liver IGF-1-deficient (LID) mouse. Contrary to deficits in controls, OVX of LID mice resulted in maintenance of cortical bone mechanical integrity primarily owing to an enhanced periosteal expansion affect on cross-sectional structure (total area and cortical width). The serum balance in LID that favors GH over IGF-1 diminished the effects of ablated ovarian function on numbers of osteoclast precursors in the marrow and viability of osteocytes within the cortical matrix and led to less endosteal resorption in addition to greater periosteal bone formation. Interactions between estrogen and the GH/IGF-1 system as related to bone remodeling provide a pathway to minimize degeneration of bone tissue structure and osteoporotic fracture. © 2010 American Society for Bone and Mineral Research

## Introduction

Insulin-like growth factor (IGF-1) is a major regulator of skeletal growth and remodeling.(1–3) In vivo, IGF-1 mediates the effects of growth hormone (GH) on longitudinal bone growth, whereas in vitro, it is a mitogen for preosteoblasts and promotes their ability to mineralize in culture.(4–6) Studies by us and others have shown that IGF-1 acts on bone via both endocrine and autocrine/paracrine pathways.(1–3,7) Endocrine regulation of bone is mediated by circulating IGF-1, which is the most prevalent peptide hormone secreted by the liver(4); autocrine/paracrine regulation depends on skeletal IGF-1 produced by osteoblasts and by stromal cells of bone marrow.(5)

One of the factors regulating the expression of IGF-1 is estrogen, a steroid hormone whose role in maintaining skeletal integrity through effects on bone remodeling has been documented extensively.(8–10) Loss of estrogen in postmenopausal women, ovariectomized animals, or men deficient in the production and/or response to estrogen in peripheral tissues results in increased bone turnover and a net increase in bone resorption over formation, resulting in reduced bone mineral density (BMD).(11–13) In addition to inhibiting production of proresorptive cytokines such as tumor necrosis factor α (TNFα), interleukin 1 (IL-1), IL-6, IL-7, and receptor activator of NF-κB (RANKL) by osteoclast-supportive bone marrow (BM) stromal cells, monocytes, and lymphocytes,(14,15) estrogen has been shown to stimulate IGF-1 expression by osteoblastic cells.(16) Moreover, the liver responds to sex steroids, and numerous clinical and animal studies have shown that sex steroids can modulate GH secretion (at the level of the pituitary) as well as its action in target tissues (i.e., liver or bone).(17) Finally, serum GH levels correlate positively with estrogen, and estrogen-replacement therapy in postmenopausal women increases serum GH, even though serum IGF-1 levels are decreased.(18,19) These findings indicate that complex interactions between the GH/IGF-1 and estrogen axes may contribute to skeletal maintenance, and suggest that treatment with GH may have potential benefit in postmenopausal bone loss.

Because estrogen can regulate circulating IGF levels via actions in both the liver and pituitary (through GH), we hypothesized that the skeletal response to ovariectomy-induced bone loss would be altered if the ability of the liver to express and produce circulating IGF-1 were limited. We tested this hypothesis using the LID mouse system generated using the Cre-lox*P* system.(20) LID mice Exhibit 75% lower circulating IGF-1 levels and three- to fourfold greater serum GH levels than controls.

## Materials and Methods

### Animals

We previously described the generation and genotyping of LID mice.(20) Briefly, LID mice were generated using the Cre-lox*P* system, whereby exon 4 of the *IGF1* gene is flanked by two lox*P* sites, and the Cre recombinase Tg is expressed specifically in the liver under the albumin enhancer-promoter sequence. PCR was used for genotype determination of littermates with specific primers and tail DNA extracts. The following primers were employed: 5′-AAA CCA CAC TgC TCg ACA TTg, 5′-AgT gAT Agg TCA CAA AgT TCC, and 5′-CAC TAA ggA gTC TgT ATT Tgg ACC to detect the WT and *IGF1* recombinant allele. The Cre recombinase Tg was detected with 5′-AAT gCT TCT gTC CgT TTg CCg gT and 5′-CCA ggC TAA gTg CCT TCT CTA CA.

Separate, nonoperated basal control groups of 12-week-old mice (*n* = 8 per group) were sacrificed at the beginning of the experiment to account for growth. Mice were caged in groups of four or five and fed *ad libitum*. Body weight (BW) and composition (see below) were measured every 4 weeks. The IACUC at the Mount Sinai School of Medicine approved all animal study protocols. All protocols requiring anesthesia used 3% inhaled isofluorane (Baxter) in 1 L/min oxygen. No mice died unintentionally over the experimental periods.

### Ovariectomy

Mice were anesthetized, the lumbar dorsum shaved bilaterally, and the exposed skin prepared for aseptic surgery. For each ovary, a single 0.7 cm dorsal flank incision penetrating the abdominal cavity was made. The exposed ovary was removed by scalpel excision, and homeostasis was achieved by hemostat pressure for 1 to 2 minutes. The incisions were closed with nonabsorbable suture. Injection (s.c.) of buprenorphine hydrochloride (0.05 mg/kg, Reckitt Benckiser PLC) provided postoperative analgesia.

### Body composition

Fat mass was assessed in live (nonanesthetized) animals using EchoMRI (3-in-1, Echo Medical Systems). This technique allows serial measurements of fat and lean mass with high levels of precision and without affecting the subjects tested. The measurement of each mouse lasted 90 seconds, and the precision of the measurement is 0.1 to 0.3 SD.

### Blood and tissue collection

Mice were anesthetized and mandibular bleeding performed to obtain serum samples. Liver, bone marrow (BM) and bones were dissected immediately after euthanasia and frozen in liquid nitrogen or fixed in 10% neutral phosphate-buffered formalin (PBF). Bones for micro-computed tomography (µCT) and mechanical testing were stored at −20°C.

### Serum hormones

#### IGF-1

Serum concentrations were measured using an RIA with a polyclonal human anti-IGF-1 Ab that has been validated previously in mice.(7) The sensitivity of measurement is 0.3 ng/mL; there is no cross-reactivity with IGF-2, the interassay CV is 4.6%, and the intraassay CV is 2.3%. Known standards were placed in each run, and pooled mouse serum from C57BL/6J mice at 16 weeks of age was used as another control for assay variation.

#### GH

Serum concentrations were determined using an RIA kit with a sensitivity of 0.02 ng/mL (National Hormone and Pituitary Program, Harbor-UCLA Medical Center, Los Angeles, CA).

#### IGFBP

Serum levels of mouse IGF-binding protein-3 (IGFBP-3) were determined with ELISA assays developed at UCLA using recombinant mouse proteins from R&D and mAb as described previously.(21)

#### µCT

Three-dimensional µCT (µCT40, Scanco Medical AG) was used to assess bone structure using a 12 µm isotropic voxel resolution, as described elsewhere.(22) All morphometric traits were computed from binarized images using direct 3D techniques that do not rely on any prior assumptions about the underlying structure.(23) Nomenclature adheres to that recommended by the Histomorphometry Nomenclature Committee of the American Society for Bone and Mineral Research.(24) For distal femoral metaphyses, trabecular bone volume (BV/TV), trabecular thickness (Tb.Th), trabecular number (Tb.N), trabecular span (Tb.Sp), and connectivity density (Conn-Dens) were quantified. Middiaphyseal morphologic traits measured were Tt.Ar, Ct.Ar, Ma.Ar, Ct.Wi, and cross-sectional moment of inertia (CSMI). The diaphyseal site corresponded to the typical location of failure during the bending tests (see below). For quality assurance, we followed the manufacturer's recommendations, which include weekly scans of a set of hydroxyapatite phantoms, weekly archiving of data and system software, and an annual maintenance and service visit by a Scanco Medical technician.

### Whole-bone mechanical testing

Long bone length was measured as the caliper distance (0.01 mm resolution) between the most proximal and distal articulating surfaces of the femoral head and condyles, respectively. The mechanical properties of middiaphyseal femurs then were quantified by loading to failure in four-point bending at 0.05 mm/s using a servo-hydraulic materials test system (Instron Corporation).(25,26) The four-point bending test allows comparison of mechanical properties as calculated from the load and displacement signals. Femurs were tested at room temperature and kept moist with PBS. Each femur was placed with the caudal surface down on two lower supports. The lower and two upper supports were set apart at 6.35 and 2.2 mm, respectively, and loading was centered over the middiaphysis. Load and displacement signals were bridge amplified, with the same gain in each test. These signals, comprising the load-deflection curves, were fed to a Gateway PC equipped with a National Instruments AT-MIO 16-bit analog-to-digital (A/D) board. The A/D board was configured as single-ended with a range of ±10 V. Digital sampling was accomplished at 100 Hz with custom-coded lab software using the LabView package (Version 6, National Instruments). Maximum signal intensity without loss of data owing to digitization was ensured by use of a protocol for similar-sized mouse femurs, and data cutoff was not observed. Load-deflection curves were analyzed with custom-coded LabView software (courtesy of Dr. Karl J Jepsen) for stiffness (the slope of the initial linear portion of the curve) and strength (maximum load).

### Bone histomorphometry

Because differences in skeletal organization and mechanical properties may arise from differences in bone formation, resorption, or both, we carried out histomorphometric assessments of these processes in the tibial middiaphysis as described in detail elsewhere.(24,25) Briefly, calcein (10 mg/kg, Sigma C-0875) labels were injected intraperitoneally at 8 and 2 days prior to euthanasia to label bone-forming surfaces (osteoblast activity). Following euthanasia, tibias were removed, fixed in 10% neutral PBF, bulk stained with Villanueva bone stain, and embedded in poly-methyl methacrylate. Embedded bones were sectioned transversely at the middiaphysis with a diamond-coated wafering saw (Leica), polished to a thickness of 30 µm with silicon carbide abrasive paper, and mounted on slides. Cortical bone indices of static bone geometry (Tt.Ar, Ct.Ar, and Ma.Ar) and osteoblast bone formation (L.Pm/B.Pm and MAR) and osteoclast bone resorption (Er.Pm/B.Pm) activity on both periosteal and endosteal surfaces were analyzed using an OsteoMeasure system (Osteometrics, Decatur, GA) on a Zeiss Axioskop microscope with fluorescence and brightfield capability.(24) Area fractions (Ct.Ar/Tt.Ar and Ma.Ar/Tt.Ar) and bone-formation rate (BFR/B.Pm, the product of MAR and L.Pm/B.Pm) were calculated.(24) A single observer blinded to the specimen identity made all measurements.

### Osteocyte apoptosis

The spatial patterns of osteocyte apoptosis were assessed in middiaphyseal femoral cortices using immunohistochemistry for apoptotic markers.(27) To detect osteocyte apoptosis, 5 µm thick paraffin sections were cut and incubated with primary Ab to cleaved (activated) caspase-3 (Cell Signaling No. 9661, at 1:50 dilution in Ab diluent, DakoCytomation, Carpinteria, CA). Sections were adhered to positively charged slides, deparaffinized, rehydrated, and then treated with 3% hydrogen peroxide to block endogenous peroxidase activity. Antigen retrieval was performed using a methanol-NaOH-based solution for 30 minutes at room temperature (DeCal Retrieval Solution, Biogenex, San Francisco, CA). Before addition of primary Ab, nonspecific tissue binding was blocked by incubating tissue sections in 10% rabbit serum in PBS for 30 minutes at room temperature. Sections were incubated with rabbit antimouse cleaved caspase-3 primary Ab to identify cells undergoing apoptosis. Sections were incubated in primary Ab overnight at 4 °C in a humidified chamber. Detection was performed using a goat antirabbit secondary Ab labeled with a horseradish peroxidase conjugate and developed with a 3,3'-diaminobenzidine (DAB) substrate chromogen system (DakoCytomation); fast-green was used as a counterstain. Apoptotic osteocytes (caspase-positive) were counted under brightfield microscopy at × 400 magnification using a 10 × 10 mm eyepiece grid reticule. Osteocytes were counted through the entire cortical width for each of eight sampling regions around the cross section.

### Liver expression of GH receptor and BP

In the circulation, GH is bound to a high-affinity binding protein (GHBP) that affects its pharmacokinetics and bioactivity. However, the biologic role of GHBP is not yet clear. In cell culture systems, GHBP antagonizes GH actions, and in animal models, administration of GHBP was shown to enhance GH bioactivity and prolong GH half-life in serum.(28) We examined expression of GHBP and GH receptor (GHR) levels in livers of all groups at baseline and 4 weeks postoperatively. One microgram of total RNA was extracted from livers and reverse-transcribed to cDNA. Real-time PCRs of GHR, GHBP, and β-actin were performed using specific primers.

### BM fluorescence-activated cell sorting

We assessed changes in the immune cell populations of BM isolates from control and LID mice at 4, 8, and 16 weeks postoperatively. We focused on two cell populations identified by specific cell surface markers: (1) B220, a marker of pre-pro-B cells, which produce membrane-bound and secreted RANKL, an essential differentiation factor for osteoclasts, and (2) CD11b, a marker of the myeloid lineage from which osteoclasts differentiate on stimulation with RANKL/M-CSF. BM cells were harvested from the femur, washed with PBS, and resuspended in staining buffer (PBS, 0.5% FBS, 0.09% sodium azide): 10^6^ cells were preincubated with rat antimouse CD16/CD32 (1 µg, BD Biosciences, San Diego, CA) for 10 minutes at 4°C to block Fc receptors. Cells were incubated for 30 minutes at 4°C with fluorescently labeled Ab: R-Phycoerythrin (PE)-c-Fms/CSF-1R (Santa Cruz Biotechnology, Santa Cruz, CA, USA), PE-Cy7-conjugated rat antimouse CD45R/B220, and Alexa Fluor 647-conjugated rat antimouse CD11b (BD Biosciences). The cells were washed with cold PBS buffer. BM cells then were resuspended in PBS containing 1% paraformaldehyde. Cell acquisition was performed in a flow cytometer (FACScan, Becton Dickinson, Franklin Lakes, NJ), and at least 10,000 events were acquired for each test. Data were analyzed with FlowJo software (Version 7.2). R-PE-conjugated mouse IgG_2b_ and Alexa Fluor 647-conjugated mouse IgG_2a_ isotype controls were used. Cells were stained with Streptavidin-FITC diluted 1:100 as control.

### Statistics

Differences in trait values among the groups and all time points were assessed individually by two-way ANOVA with interaction. The type I error rate (α) was set at 0.05. Differences between groups at individual time points were compared using post hoc Bonferroni-adjusted multiple comparisons (SYSTAT Software, SPSS Science, Chicago, IL).

## Results

### Changes in body adiposity in control and LID mice

**Fig. 1:**
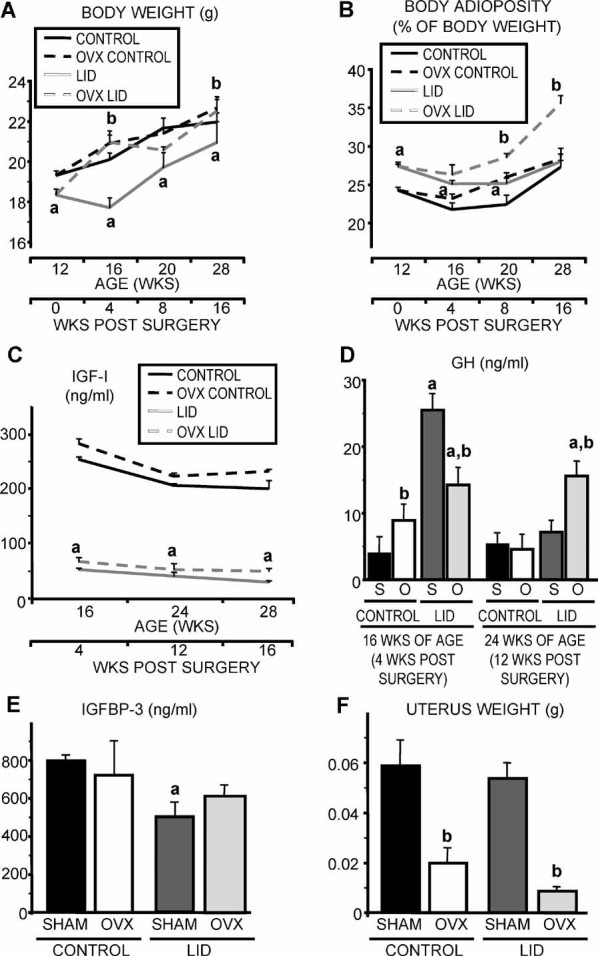
The effects of OVX on body composition and serum levels of IGF-1, IGFBP-3, and GH in 12-week-old (basal) control and LID female mice. (*A*) BW measured from 12 to 28 weeks of age. (*B*) Body adiposity assessed by MRI. (*C*) Serum IGF-1 determined by RIA. (*D*) Serum levels of GH in sham-operated (S) mice and mice after OVX (O) determined by ELISA. (*E*) Serum IGFBP-3 levels determined by ELISA. (*F*) Uterus weight at 4 weeks after surgery, *n* = 8 mice per group, except sham-operated control mice. where *n* = 4. For longitudinal measurements (*A–D*), *n* = 8 to 47 mice per group. Data are represented as mean ± SEM at *p* < .05. a = LID versus sham-operated control or basal mice of same age group (significant genotype effects); b = versus sham-operated mice of same genotype and age group (significant OVX effects).

Intact LID female mice had a lower BW than control mice at 12 weeks of age and remained smaller at all ages examined (Fig. 1*A*). OVX did not alter body mass in control mice but resulted in substantial weight gains in the LID mice so that their weights at 4 to 16 weeks aftert OVX did not differ from those of age-matched controls. MRI verified that the increased BW in LID mice following OVX was due to increased body fat mass (see Fig. 1B).

### Serum levels of IGF-1, IGFBP-3, and GH

LID mice exhibit 75% lower serum IGF-1 levels than control mice at birth, and this difference persists throughout life with no evident changes in *IGF1* gene expression in other tissues.(20) OVX did not affect serum IGF-1 levels in LID or control mice (see Fig. 1*C*). In contrast, serum GH levels responded differently to OVX in control and LID mice. At 16 weeks of age (4 weeks postoperatively), sham-operated LID mice had roughly sixfold greater serum GH levels than controls, but this difference was no longer evident at 24 weeks, indicating an age-related decrease in serum GH levels (see Fig. 1*D*). In OVX mice, however, GH levels were about 1.6-fold greater in LID mice than in controls at 16 weeks (4 weeks after OVX), and at 24 weeks of age (12 weeks after OVX), GH levels remained elevated, resulting in a roughly threefold increase relative to control mice. Alongside a lower serum IGF-1 level, IGFBP-3, the main carrier of IGF-1 in circulation, also was lower in LID mice than in controls, as expected given the direct dependency of IGFBP-3 on IGF-1.(18) This difference persisted at 4 weeks postoperatively (see Fig. 1*E*). As expected, OVX reduced uterine mass in both control and LID mice and to a similar extent (∼75%) versus sham-operated animals (see Fig. 1*F*).

### Structural and mechanical properties

**Fig. 2:**
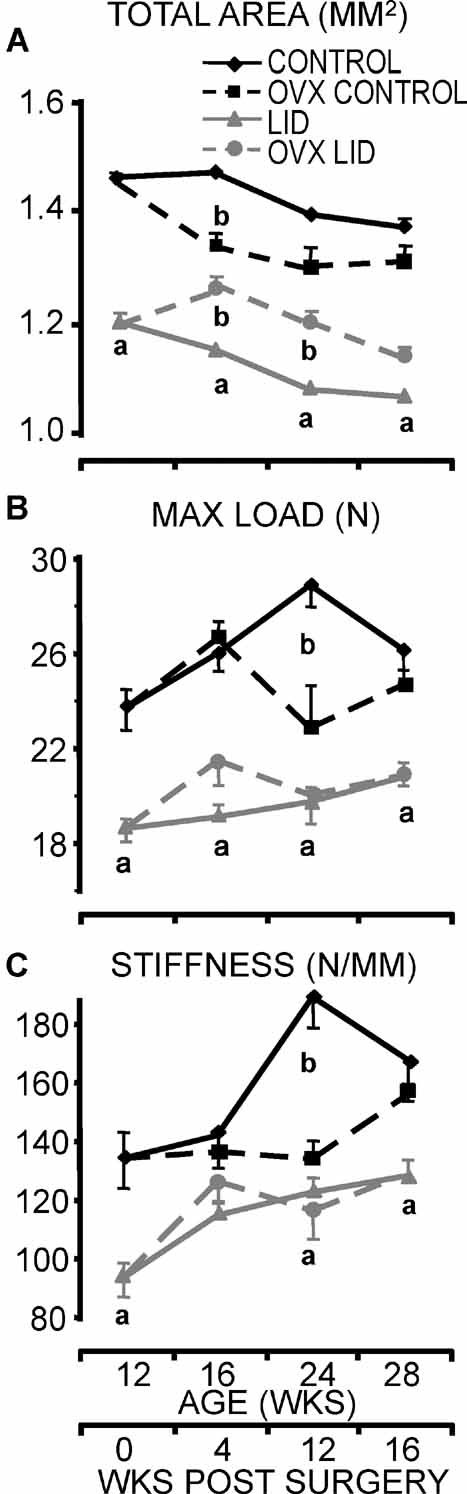
Femoral midshaft structural and biomechanical properties determined by µCT and four-point bend test, respectively. (*A*) Total area at the femoral midshaft. Maximum load (*B*) and stiffness (*C*) sustained by the femoral midshaft. Data are represented as mean ± SEM. Group sizes were basal: control (*n* = 7), LID (*n* = 8); 16 weeks: sham-operated control (*n* = 4), OVX control (*n* = 7), sham-operated LID (*n* = 8), OVX LID (*n* = 8); 24 weeks: sham-operated control (*n* = 7), OVX control (*n* = 7), sham-operated LID (*n* = 7), OVX LID (*n* = 8); and 28 weeks: sham-operated control (*n* = 10), OVX control (*n* = 15), sham-operated LID (*n* = 9), OVX LID (*n* = 19). *p* < .05. a = LID versus sham-operated control or basal mice of same age group (significant genotype effects); b = versus sham-operated mice of the same genotype and age group (significant OVX effects).

The structural consequences of serum IGF-1 and estrogen deficiencies on long bones were revealed by µCT (Fig. 2*A* and Table 1). Femurs of LID mice were thinner than controls at all ages based on Tt.Ar at the middiaphysis (see Fig. 2*A*). Additionally, LID mice exhibited a smaller cortex thickness (Ct.Wi). The effects of OVX also differed between control and LID mice. OVX led to a decrease in Tt.Ar and the expected cortical thinning (smaller Ct.Wi) in controls, whereas expansion of the periosteal envelope in LID mice shortly after OVX resulted in larger Tt.Ar and maintenance of Ct.Wi.

**Table 1:** Indices From µCT of Femurs From Control and LID Mice at Baseline (12 Weeks of Age) and at 4, 12, and 16 Weeks After Surgery

Age	Location	Variable	Control	LID
12 weeks, basal	Distal femur	BV/TV (%)	15 ± 0.69	12 ± 0.66^a^
		Conn-Dens (1/mm^3^)	134 ± 9.1	121 ± 10.2
		Tb.N (1/mm)	4.4 ± 0.09	4.2 ± 0.11
		Tb.Th (µm)	51 ± 0.49	47 ± 0.52^a^
		Tb.Sp (µm)	225 ± 5.5	240 ± 6.6
	Mid-diaphysis femur	Ct.Ar (mm^2^)	0.61 ± 0.02	0.48 ± 0.01^a^
		Ma.Ar (mm^2^)	0.83 ± 0.01	0.71 ± 0.02^a^
		Ct.Ar/Tt.Ar (%)	42 ± 0.74	40 ± 0.48
		Ct.Wi (µm)	163 ± 3.90	140 ± 1.86^a^
		CSMI (mm^4^)	0.22 ± 0.008	0.14 ± 0.006^a^

	Location	Variable	Sham control	OVX control	Sham LID	OVX LID
**1**6 weeks	Distal femur	BV/TV (%)	11 ± 1.25^c^	9.9 ± 0.48	9.7 ± 0.50	8.4 ± 0.23
		Conn-Dens (1/mm^3^)	81.0 ± 14.3^c^	65.0 ± 7.0	83.7 ± 7.9^c^	69.7 ± 3.6
		Tb.N (1/mm)	3.7 ± 0.19^c^	3.5 ± 0.09	3.5 ± 0.10^c^	3.2 ± 0.08
		Tb.Th (µm)	53 ± 1.07	48 ± 1.21^b^	47 ± 0.29^a^	47 ± 0.73
		Tb.Sp (µm)	273 ± 14.1	314 ± 16.7	293 ± 8.3	324 ± 8.3
	Mid-diaphysis femur	Ct.Ar (mm^2^)	0.67 ± 0.01	0.61 ± 0.03^b^	0.48 ± 0.01^a^	0.53 ± 0.02
		Ma.Ar (mm^2^)	0.79 ± 0.01	0.77 ± 0.03	0.66 ± 0.01^a^	0.72 ± 0.02
		Ct.Ar/Tt.Ar (%)	46 ± 0.53^c^	44 ± 0.81	42 ± 0.55^a^	42 ± 0.63
		Ct.Wi (µm)	179 ± 3.17^c^	169 ± 5.10^b^	145 ± 2.07^a^	153 ± 3.66
		CSMI (mm^4^)	0.23 ± 0.006	0.17 ± 0.028^b^	0.13 ± 0.004^a^	0.16 ± 0.010
24 weeks	Distal femur	BV/TV (%)	6.5 ± 0.43^c^,^d^	8.7 ± 1.34	7.7 ± 0.87	4.8 ± 0.43^a^^b^,^d^
		Conn-Dens (1/mm^3^)	19.2 ± 3.2^c^,^d^	53.3 ± 14.5^b^,^d^	51.6 ± 9.1^a^^c^,^d^	25.3 ± 4.1^a^,^d^
		Tb.N (1/mm)	2.8 ± 0.09^c^,^d^	2.9 ± 2.8	3.0 ± 0.18^c^	2.4 ± 0.07^b^,^d^
		Tb.Th (µm)	53 ± 1.20	50 ± 1.02	45 ± 0.53^a^	45 ± 0.57^a^
		Tb.Sp (µm)	367 ± 12.7	364 ± 29.6	342 ± 22.8^c^	424 ± 12.3^b^,^d^
	Mid-diaphysis femur	Ct.Ar (mm^2^)	0.67 ± 0.01	0.59 ± 0.02^b^	0.51 ± 0.01^a^	0.52 ± 0.02^a^
		Ma.Ar (mm^2^)	0.71 ± 0.01^c^	0.71 ± 0.02	0,5 ± 0.01^a^^c^,^d^	0.67 ± 0.01^b^
		Ct.Ar/Tt.Ar (%)	48 ± 0.42^c^	46 ± 0.34^b^	48 ± 0.57^c^,^d^	44 ± 0.60^b^
		Ct.Wi (µm)	188 ± 2.24^c^	169 ± 3.40^b^	164 ± 3.32^a^^c^,^d^	155 ± 4.25^a^
		CSMI (mm^4^)	0.21 ± 0.005	0.19 ± 0.014	0.13 ± 0.004^a^	0.15 ± 0.010
28 weeks	Distal femur	BV/TV (%)	5.9 ± 0.46^c^,^d^	5.8 ± 0.53d,e	6.4 ± 0.54^d^	5.3 ± 0.28^d^
		Conn-Dens (1/mm^3^)	19.0 ± 2.9^c^,^d^	22.9 ± 3.5^d^,^e^	40.5 ± 5.7c,d,e	27.7 ± 2.4^d^
		Tb.N (1/mm)	2.6 ± 0.06^c^,^d^	2.3 ± 0.06d,e	2.7 ± 0.13^c^,^d^	2.1 ± 0.09^b^,^d^
		Tb.Th (µm)	52 ± 1.28	50 ± 0.59	46 ± 0.88^a^	46 ± 0.43^a^
		Tb.Sp (µm)	387 ± 12.1^c^,^d^	437 ± 11.1d,e	386 ± 26.5^c^,^d^	491 ± 21.5^b^,d,e
	Mid-diaphysis femur	Ct.Ar (mm^2^)	0.65 ± 0.02	0.61 ± 0.01	0.49 ± 0.01^a^	0.49 ± 0.00^a^
		Ma.Ar (mm^2^)	0.70 ± 0.02^c^,^d^	0.71 ± 0.01	0,5 ± 0.01^a^^c^,^d^	0.64 ± 0.01^a^^b^,^d^
		Ct.Ar/Tt.Ar (%)	48 ± 0.50^c^	46 ± 0.44^b^,^d^	47 ± 0.41^c^,^d^	44 ± 0.34^a^^b^
		Ct.Wi (µm)	185 + 3.70^c^	173 ± 2.41^b^	159 ± 2.12^a^c,d	151 ± 1.17^a^
		CSMI (mm^4^)	0.21 + 0.012	0.19 ± 0.007	0.12 ± 0.004^a^	0.14 ± 0.008^a^

Data are presented as mean ± SEM. Sample sizes were basal: control (*n* = 8), LID (*n* = 8); 16 weeks: sham control (*n* = 4), OVX control (*n* = 8), sham LID (*n* = 8), OVX LID (*n* = 8); 24 weeks: sham control (*n* = 7), OVX control (*n* = 7), sham LID (*n* = 7), OVX LID (*n* = 8); and 28 weeks: sham control (*n* = 10), OVX control (*n* = 15), sham LID (*n* = 9), OVX LID (*n* = 19) at *p* < .05. Ct.Wi = cortical width; CSMI = cross-sectional moment of inertia; Conn-Dens = connectivity density.

a Versus control mice of the same surgical and age group (significant genotype effects),

b Versus sham-operated mice of the same genotype and age group (significant OVX effects),

c,d,e Versus. 12, 16, or 24 weeks, respectively, of same surgical and genotype group (significant age effects).

Mechanical testing and structural data were consistent. The thinner femoral diaphyses of LID mice had lower strength (maximum load) than controls at all ages (see Fig. 2*B*). Likewise, a thinner middiaphysis observed after OVX of control mice was associated with lower strength and stiffness at 24 weeks of age (see Fig. 2*B*, *C*). Conversely, OVX of LID mice did not result in reduced mechanical properties.

### Bone resorption and formation

**Fig. 3:**
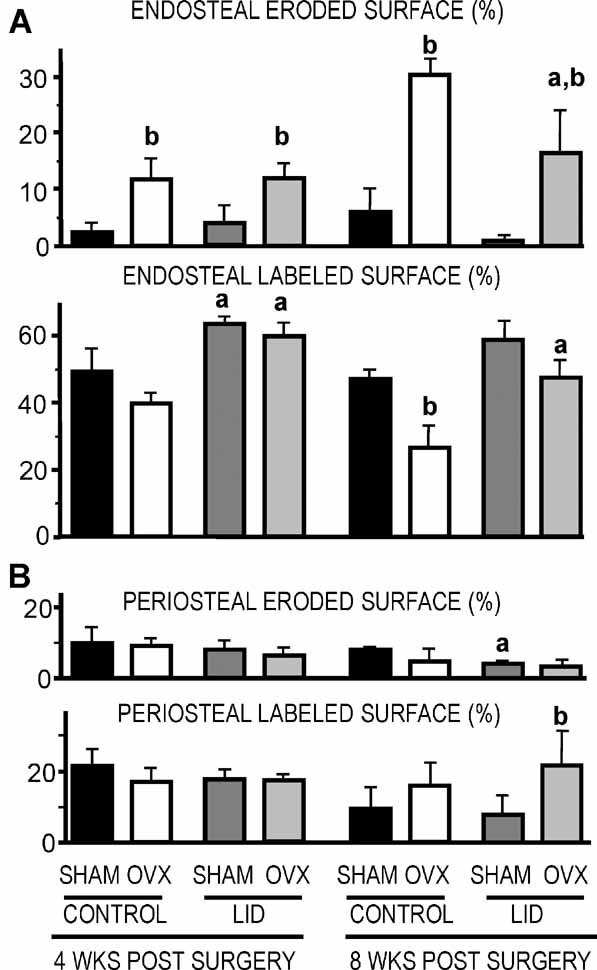
Histomorphometry at the tibial midshaft of control and LID mice at 4 and 8 weeks postoperatively. Endosteal (*A*) and periosteal (*B*) eroded surface and labeled surface expressed as perimeter fractions (Er.Pm/B.Pm and L.Pm/B.Pm). Sample sizes were *n* = 4 per group, except for 20-week-old (8 weeks after surgery) sham-operated groups, which were *n* = 3 per group. Data are represented as mean ± SEM at *p* < .05. a = versus control mice of same surgical and age group (significant genotype effects); b = versus sham-operated mice of the same genotype and age group (significant OVX effects).

Differences between control and LID mice in structural features and responses to OVX measured by histomorphometry were comparable with those observed by µCT (Fig. 3 and Table 2). In control mice, the eroded (resorbing) fraction (Er.Pm/B.Pm) of the endosteal surface increased with time after OVX. In LID mice, endosteal resorption also increased, although to a lesser degree than seen in control mice. In both control and LID sham-operated mice, there was no change in endosteal bone formation (L.Pm/B.Pm) over time. However, in control mice, OVX caused a substantial decrease in endosteal formation that was not seen in LID mice at 8 weeks after surgery (see Fig. 3*A*). While differences in resorption were not observed on the periosteal surface, L.Pm/B.Pm and, consequently, BFR were significantly greater after OVX in LID mice only (see Fig. 3*B* and Table 2).

**Table 2:** Indices From Histomorphometry of Cortical Bone at the Tibial Midshaft 4 and 8 Weeks After Ovariectomy (16 and 20 Weeks of Age)

Age	Variable	Sham control	OVX control	Sham LID	OVX LID
16 weeks	Tt.Ar (mm^2^)	0.79 ± 0.04	0.86 ± 0.02	0.65 ± 0.02^a^	0.71 ± 0.04^a^
	Ma.Ar/Tt.Ar (%)	21 ± 1.8	29 ± 2.9^b^	19 ± 0.02	20 ± 1.4^a^
	Endosteal surface: Tt.Ar (mm^2^)				
	Er.Pm/B.Pm (%)	2.4 ± 1.9	12 ± 3.7^b^	4.3 ± 3.0	12 ± 2.7^b^
	L.Pm/B.Pm (%)	49 ± 7.4	40 ± 3.0	64 ± 2.3^a^	60 ± 4.0^a^
	MAR (µm/d)	1.08 ± 0.11	0.94 ± .09^b^	1.11 ± 0.06^a^	1.08 ± 0.12
	BFR/B.Pm (µm/d*100)	52.9 ± 9.7	36.9 ± 4.5^b^	70.4 ± 4.4^a^	63.9 ± 5.6^a^
	Periosteal surface:				
	Er.Pm/B.Pm (%)	9.8 ± 4.4	8.9 ± 1.5	7.9 ± 2.6	6.0 ± 2.1
	L.Pm/B.Pm (%)	21 ± 4.7	17 ± 4.2	18 ± 2.0	18 ± 1.6
	MAR (µm/d)	0.06 ± 0.07	0.42 ± 0.19^b^	0.20 ± 0.15	0.42 ± 0.21
	BFR/B.Pm (µm/d*100)	2.00 ± 2.31	8.52 ± 4.55^b^	3.75 ± 2.53	7.29 ± 4.29
20 weeks	Tt.Ar (mm^2^)	0.89 ± 0.01	0.76 ± 0.01^b^	0.74 ± 0.08^a^	0.67 ± 0.01
	Ma.Ar/Tt.Ar (%)	27 ± 2.4	31 ± 2.5	29 ± 3.4	24 ± 0.4^a^
	Endosteal surface: Tt.Ar (mm^2^)				
	Er.Pm/B.Pm (%)	6.0 ± 4.4	30 ± 2.6^b^	0.88 ± 1.1	17 ± 7.4^a^^b^
	L.Pm/B.Pm (%)	47 ± 3.8	27 ± 6.3^b^	59 ± 6.6^a^	48 ± 4.9^a^
	MAR (µm/d)	0.88 ± 0.10	0.37 ± 0.17^b^	0.97 ± 0.08	0.62 ± 0.17^b^
	BFR/B.Pm (µm/d*100)	41.8 ± 7.2	11.0 ± 6.0^b^	57.4 ± 4.0^a^	28.4 ± 6.7^a^^b^
	Periosteal surface:				
	Er.Pm/B.Pm (%)	8.1 ± 1.2	5.0 ± 3.4	4.1 ± 1.5^a^	3.5 ± 2.4
	L.Pm/B.Pm (%)	9.9 ± 4.2	16 ± 3.7	7.6 ± 3.5	22 ± 9.7^b^
	MAR (µm/d)	ND	0.31 ± 0.20	0.15 ± 0.18	0.35 ± 0.27
	BFR/B.Pm (µm/d*100)	ND	4.5 ± 3.0	1.5 ± 1.8	11.6 ± 8.3^b^

Double labels were not detected (ND) on the periosteal surface of 20-week sham control mouse tibias. Data are presented as mean ± SEM. Sample sizes were 16 weeks: *n* = 4 per group; and 20 weeks: sham control (*n* = 3), OVX control (*n* = 4), sham LID (*n* = 3), OVX LID (*n* = 4) at *p* < .05.

a Versus control mice of the same surgical and age group (significant genotype effects),

b Versus sham-operated mice of the same genotype and age group (significant OVX effects).

### Ovariectomy-induced osteocyte apoptosis

**Fig. 4:**
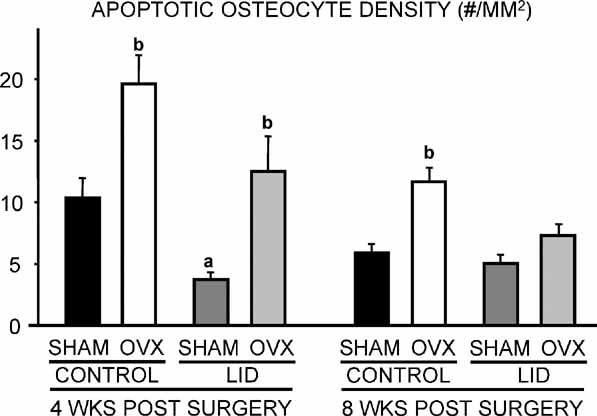
Apoptotic osteocyte density was increased after OVX and was lower in LID mice. Osteocyte apoptosis was assessed by immunohistochemistry using caspase-3 Ab on 5 µm paraffin sections of the femoral midshaft. Sample sizes were *n* = 4 per group, except for the 20-week-old (8 weeks after surgery) sham-operated groups, that were *n* = 3 per group. Data are represented as mean ± SEM at *p* < .05. a = sham-operated LID mice versus sham-operated control mice of same age group (significant genotype effects); b = versus sham-operated mice of same genotype and age group (significant OVX effects).

We detected a twofold increase in osteocyte apoptosis based on immunohistochemical staining for activated caspase-3 in control mice 4 and 8 weeks after OVX (Fig. 4). In LID mice, we observed a similar increase in apoptotic osteocytes 4 weeks aftert OVX, but at 8 weeks after OVX, the number of apoptotic osteocytes was comparable with that in sham-operated control mice.

### OVX increased GH sensitivity in LID mice

**Fig. 5:**
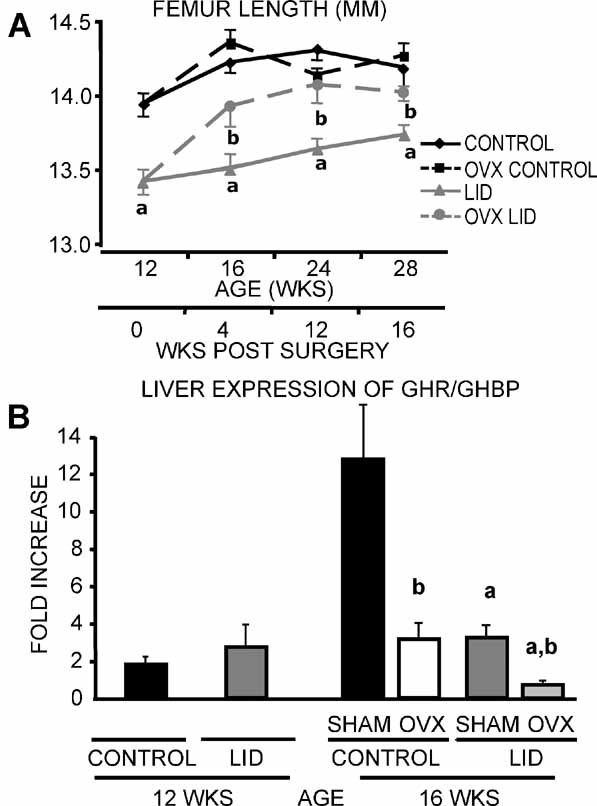
OVX increased GH sensitivity in LID mice. (*A*) Femur length was measured by caliper (*n* = 4 to 19 per group). (*B*) Real-time PCR analysis of GHBP expression in liver (*n* = 4 per group). Data are represented as mean ± SEM at *p* < .05. a = versus control mice of same surgical and age group (significant genotype effects); b = versus sham-operated mice of the same genotype and age group (significant OVX effects).

Femoral length, which depends strongly on GH activity, was not affected by OVX in control mice at 4, 12, and 16 weeks following surgery (Fig. 5*A*). However, LID mice experienced a significant increase (∼4%) in femoral length. This suggests that in the face of reduced serum IGF-1 levels, LID mice experience an increase in GH activity in bones following OVX.

Real-time PCR revealed a decrease in GHBP/GHR expression in response to OVX in both control and LID mice (see Fig. 5*B*).

### LID mice show reduced numbers of lymphoid (B220) and myeloid (CD11b) cells in bone marrow

**Fig. 6:**
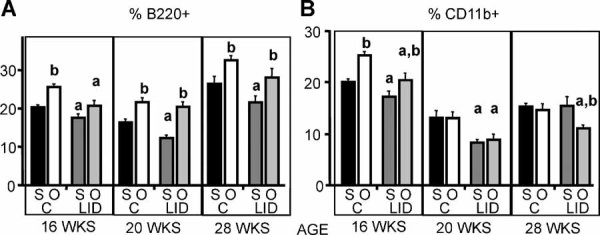
FACS analysis of cells derived from femoral marrow of control (C) and LID mice at the indicated ages after sham operation (S) or OVX (O) surgery at 12 weeks of age. B220+ cells (*A*) and CD11b+ cells (*B*). Data are represented as mean ± SEM of *n* > 6 mice per group at *p* < .05. a = versus control mice of same surgical and age group (significant genotype effects); b = versus sham-operated mice of the same genotype and age group (significant OVX effects).

LID mice exhibited significant reductions in B220+ and CD11b+ cells at all time points after surgery (Fig. 6). These data are in accordance with our previous finding of reduced resorption in LID mice after OVX.

## Discussion

**Fig. 7:**
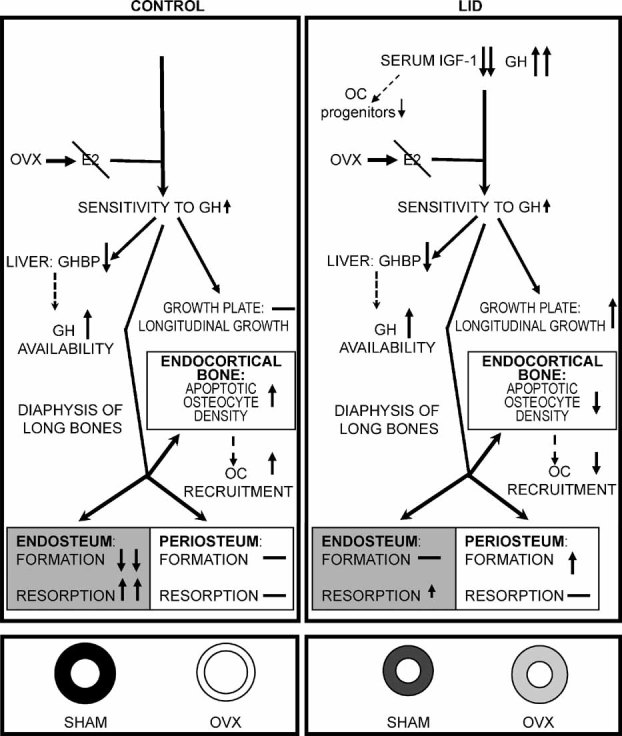
The effects of serum IGF-1 deficiency and estrogen deprivation on bone. Serum IGF-1 deficiency in LID mice results in an increase in serum GH levels. Additionally, reductions in serum IGF-1 lead to a decrease in osteoclast (OC) progenitors through as yet undefined mechanism(s). Estrogen deprivation in the state of IGF-1 deficiency leads to an increase in the sensitivity to GH, as reflected by increased linear growth and decreased production of GHBP by the liver. The increase in GH may protect osteocytes from OVX-induced apoptosis, leading to reduced recruitment of OC, inhibition of resorption at the endosteal surface, and increased formation at the periosteal surface.

The results of this study clearly demonstrate that the skeletal response to estrogen deprivation can be modulated to a large extent by alterations in the GH/IGF-1 regulatory axis, specifically by changes in the circulating levels of IGF-1 and GH. Control and LID mice exhibited marked differences in both bone resorption and formation that were associated with changes in osteocyte apoptosis as well as the content of immune-related cells in BM (Fig. 7). In addition, we observed a surprising increase in longitudinal femur growth of LID mice following OVX, an effect that may be directly attributable to increased circulating GH levels in the face of reduced circulating IGF-1 in LID mice.

Not surprisingly, as reported previously by our group, LID mice show increased GH throughout adolescence and into adulthood owing to impaired feedback inhibition by circulating IGF-1.(20) An additional and well-recognized modulator of GH is estrogen. However, estrogen effects on GH have been inconsistent, with studies showing either stimulation or inhibition. Oral estrogens inhibit the actions of GH in the liver. Clinically, this effect is observed in a number of different conditions, including estrogen supplementation in girls with delayed puberty and constitutional tall stature and postmenopausal or hypopituitary women.(29–31) In GH-deficient patients, serum IGF-1 levels are lower in females than in males, and when given GH-replacement therapy, females require a higher dose of GH to get an increase in serum IGF-1 levels similar to males.(32) Thus there appears to be GH resistance to exogenous and endogenous estrogen. We found increased bone apposition, length, and width to occur principally within the first 4 weeks of OVX in LID mice. However, during this period, GH levels dropped. Estrogen is known to modify peripheral responses to GH.(33) Therefore, we postulate that in the absence of estrogen, LID mice have increased sensitivity to GH. Centrally, we found that OVX resulted in a decreased GHR/GHBP expression in the liver. Together these data suggest that in the absence of estrogen there is an increased response to and increased bioavailability of GH in serum of LID mice.

Our data demonstrate that dysregulation of the GH/IGF-1–estradiol axis has consequences for the mechanical integrity of the diaphysis. In 12-week-old LID mice, biomechanical properties and cross-sectional morphology were different than in age-matched controls. Biomechanical properties, including maximum load, are indicators of resistance to fracture and normally are directly related to BW and/or lean mass. However, this relationship is confounded with the loss of estrogen, such as at female menopause, when BW and adiposity increase, whereas lean mass, bone mass, and biomechanical properties decrease, resulting in an increased risk of fracture.(34) OVX of LID mice was associated with greater BW and adioposity compared with sham-operated LID mice. After OVX, LID mice did not exhibit reduced diaphyseal cortical biomechanical properties, in agreement with greater midshaft structural properties (Tt.Ar and Ct.Wi). This was in sharp contrast to the normal changes observed after depletion of estrogen in control mice (see Fig. 7). Femoral Tt.Ar remained greater in LID mice with OVX than sham-treated LID mice for up to 16 weeks (*p* = .06) after surgical intervention, the last time point examined.

Data on the biomechanical consequences of an impaired GH/IGF-1 axis had not been reported previously. However, studies in adult dogs and rats show that GH treatment can increase bone mass in a dose-dependent manner.(35,36) Additionally, exogenous administration of recombinant human GH into aged rats with OVX prevents further trabecular bone loss and increases cortical bone mass above pretreatment values.(37–39) However, these previous exogenous GH studies did not attempt to separate out effects of other hormones or explore the cellular mechanisms driving increased bone apposition; the exogenous administration of either IGF-1 or GH increases circulating levels of GH or IGF-1, respectively. In our LID mouse model with high baseline GH, increased cortical bone apposition and mechanical integrity occurred only after OVX and without a significant alteration in the low level of circulating IGF-1. Combined with the previous body of work, these results argue for a direct peripheral effect of GH on bone formation. Importantly, these new data also suggest that starting GH hormone treatment prior to loss of estrogen should be further explored for protective effects on the skeleton.

There is an age-dependent uncoupling of estradiol regulation of the GH/IGF-1 axis. Studies in adult and adolescent female rhesus monkeys with OVX show that estradiol stimulates GH and IGFBP-3. Yet an increase in serum IGF-1 is restricted to adolescence, so in postpubertal young-adult females estradiol reduces serum IGF-1.(40) In this current study we chose an age for OVX at which mice were not growing rapidly (12 weeks). Additionally, we included basal groups as controls for age-related differences. The pubertal growth spurt was nearly complete, as evidenced by the low rate of longitudinal growth over our 16-week experimental period (femur ∼2%). However, BW was increased, whereas serum GH was decreased dramatically without any intervention. In adult mice, these differences are indicative of overall aging. However, we cannot rule out that OVX of older LID mice might not lead to the same suppression of endosteal osteoclast activity and enhancement of osteoblast activity as observed in this study because the physiologic milieu (stem cells, growth factors, etc.) changes with age and could affect bone resorption and formation. In a study with *GH Tg* mice, which express GH under the metallothionein promoter, increased tibial and vertebral bone volumes were revealed.(41) OVX of these mice resulted in bone loss, suggesting that the GH effects in bone depend on the presence of sex steroids, although OVX was performed at 4 weeks of age, a period of rapid growth.

Our histomorphometry data indicate that reduced serum IGF-1 was associated with reduced resorption and greater formation surface. We speculate that these resorptive and formative effects were due mainly to less circulating IGF-1 and more circulating GH, respectively. Our speculations are based on the effects of serum IGF-1 in promoting myeloid precursor populations(42,43) and GH in increasing bone growth and mass via effects on osteoblasts.(4,38,39,41) However, owing to their complex and integral relationship, as discussed previously, the separation of GH and IGF-1 effects in vivo continues to be actively pursued in many laboratories, including our own, where we are approaching the problem by the generation of novel inducible gene-expression mouse models. In both LID and control mice, the largest effect of OVX on resorption activity was measured on the endosteal surface (see Fig. 3). However, the observed loss of stiffness and strength owing to OVX in control mice is largely accounted for by differences in periosteal-associated morphology and consistent with smaller cross-sectional size (Tt.Ar and Ct.Wi). Conversely, periosteal bone formation was enhanced after OVX of LID mice (see Fig. 7), and this cellular activity provided for unaltered or increased mechanical properties.

In addition to estrogen, adioposity is a powerful regulator of GH.(33) OVX was associated with a significantly greater BW and greater adiposity in the LID mice after 4 weeks that persisted to the last time point examined (16 weeks after OVX). BW differences may affect bone structure and can be obviated by pair feeding. However, the BW increase in LID mice appears to occur regardless of caloric intake; food intake after surgery was not different between control and LID mice, irrespective of ovarian status (data not shown). Body adiposity remained increased owing to OVX only in LID mice at 16 weeks postoperatively, and the gap appeared to be widening along with differences in BW. OVX of sexually mature and otherwise normal mice does not normally lead to increased BW.(22) We speculate that most of the initial increase in BW in LID mice measured at 4 weeks after OVX was due to longitudinal growth, an effect that may be directly attributable to increased circulating GH levels in the face of reduced circulating IGF-1. Mainenance of this greater BW also likely was due to the greater adiposity in LID mice. It is conceivable that part of the effects we observed in bones of LID mice with OVX is mediated through leptin because body adiposity correlates positively with serum leptin levels, and leptin has been identified as a potent inhibitor of bone formation in mice.(44,45) However, this is unlikely because we observed greater bone formation in LID mice compared with controls following OVX.

The cells of the immune system (B- and T-lymphocytes) also play an important role in regulating bone turnover, and IGF-1 is a regulator of immune function.(42,43,46,47) Progenitors of osteoclasts arise from the same hematopoetic lineage as B and T cells.(43,47) Osteoclast differentiation acts through an axis that includes the RANK receptor on these precursors.(46) Therefore, and based on previous work demonstrating that male LID mice exhibit decreases in the myeloid cell population in BM, we hypothesized that the chronic reduction in circulating IGF-1 would affect remodeling and reduce bone resorption through changes in the immune regulatory system.(48) Indeed, we found a decrease in B220+ cells, which express a membrane-bound and secreted RANKL. Additionally, we revealed a decrease in CD11b+ cells, which are of the myeloid lineage and potentially can differentiate to osteoclasts. Together these findings suggest that IGF-1 deficiency leads to reductions in progenitor cell populations that are important for osteoclast and osteoblast proliferation and differentiation.

Cell signaling for osteoclast recruitment also affects local bone resorption. Osteocytes, which are buried in the bone matrix, maintain bone mass principally by serving as a mechano- or hormonal-sensor in bone. Animal studies suggest that osteocyte apoptosis occurs in response to OVX(49) and that apoptotic osteocytes initiate osteoclast recruitment, eventually leading to bone resorption.(11,27,50–52) Consistent with this, we found that in both control and LID mice there was a twofold increase in apoptotic osteocyte density 4 weeks after OVX. However, while in control mice we could detect this increase even 8 weeks after OVX, LID mice showed no difference in apoptotic osteocyte density between sham surgery and OVX. Moreover, regardless of ovarian status, LID mice exhibited a significantly lower apoptotic osteocyte density, possibly owing to IGF-1-independent antiapoptotic actions of increased GH.(53) Additional work will be required to define this potential GH-dependent protective pathway. In vitro studies can be particularly useful in defining the mechanisms that regulate apoptosis in osteocytes in response to changes in the levels of endocrine/paracrine regulators. However, additional in vivo studies also will be necessary to define the local levels of GH, IGF-1, and the relevant binding proteins and their changes in response to estrogen depletion. Nonetheless, our findings suggest that the low osteoclastic activity in LID mice results from both a reduced osteoclast recruitment signal (osteocyte apoptosis) and a low number of osteoclast progenitors (see Fig. 7).

Not only are our findings important to understanding the role of circulating IGF-1 and GH in mice, but they also appear similar to clinical observations and may have implications for influencing fracture risk. Estrogen loss is associated with endosteal bone loss that in long bones requires compensation by periosteal new bone formation to maintain mechanical properties and help to prevent fracture.(9,34,54–56) In states of low serum IGF-1 and estrogen deficiency (i.e., older postmenopausal women), increases in GH levels could decrease bone loss and enhance structure, including increases in periosteal bone formation, overall size, and mechanical integrity.
